# Epigenetic immune monitoring for COVID-19 disease course prognosis

**DOI:** 10.3389/fimmu.2023.1107900

**Published:** 2023-03-14

**Authors:** Björn Samans, Marta Rosselló Chornet, Araceli Rosselló Chornet, Janine Jung, Konstantin Schildknecht, Laura Lozza, Lourdes Alos Zaragoza, Javier Hernández Laforet, Nina Babel, Sven Olek

**Affiliations:** ^1^ Ivana Türbachova Laboratory for Epigenetics, Epiontis, Precision for Medicine GmbH, Berlin, Germany; ^2^ Charité – Universitätsmedizin Berlin, corporate member of Freie Universität Berlin and Humboldt Universität zu Berlin, Berlin, Germany; ^3^ Department of Anesthesiology and Resuscitation, Consortium General University Hospital of Valencia, Valencia, Spain; ^4^ Center for Translational Medicine, Medical Clinic 1, Marien Hospital Herne, University Hospitals of the Ruhr-University of Bochum, Herne, Germany

**Keywords:** COVID-19, SARS-CoV-2, epigenetic qPCR, immune monitoring, lymphopenia, disease prognosis

## Abstract

**Background:**

The course of COVID-19 is associated with severe dysbalance of the immune system, causing both leukocytosis and lymphopenia. Immune cell monitoring may be a powerful tool to prognosticate disease outcome. However, SARS-CoV-2 positive subjects are isolated upon initial diagnosis, thus barring standard immune monitoring using fresh blood. This dilemma may be solved by epigenetic immune cell counting.

**Methods:**

In this study, we used epigenetic immune cell counting by qPCR as an alternative way of quantitative immune monitoring for venous blood, capillary blood dried on filter paper (dried blood spots, DBS) and nasopharyngeal swabs, potentially allowing a home-based monitoring approach.

**Results:**

Epigenetic immune cell counting in venous blood showed equivalence with dried blood spots and with flow cytometrically determined cell counts of venous blood in healthy subjects. In venous blood, we detected relative lymphopenia, neutrophilia, and a decreased lymphocyte-to-neutrophil ratio for COVID-19 patients (n =103) when compared with healthy donors (n = 113). Along with reported sex-related differences in survival we observed dramatically lower regulatory T cell counts in male patients. In nasopharyngeal swabs, T and B cell counts were significantly lower in patients compared to healthy subjects, mirroring the lymphopenia in blood. Naïve B cell frequency was lower in severely ill patients than in patients with milder stages.

**Conclusions:**

Overall, the analysis of immune cell counts is a strong predictor of clinical disease course and the use of epigenetic immune cell counting by qPCR may provide a tool that can be used even for home-isolated patients.

## Introduction

SARS-CoV-2 has infected more than 627 million people and claimed more than six million deaths worldwide (1). To mitigate the risk of transmission, many countries forced patient isolation at home. This, in turn prevents (blood draw for) patient monitoring and thus creates its own challenges, including wrongly timed hospital admissions, without appropriate clinical or laboratory analyses contributing to hospital overload. The pandemic has forcefully demonstrated the limits even of advanced health systems and their response to diseases with high transmissibility and clinical severity (2). Approaches to address these challenges may include laboratory-based disease monitoring without physical presence of medical professionals at sample requisition, mitigating risk of dissemination and providing early warnings of severe disease courses.

Lymphopenia and lymphocyte-to-neutrophil ratio (LNR) correlate with severe COVID-19 course (3, 4) as well as with the course of other viral infections, including influenza or measles (5–7). T cell lymphopenia often signifies a more severe course of disease (8–10). Given this, quantification of immune cell frequencies is an important component of predicting disease course. Cell-specific quantification is generally performed by flow cytometry and requires viable cells, and thus patients’ direct contact with medical professionals. Fresh samples must be processed immediately to ensure valid measurements. Quantitative real-time PCR (qPCR)-based epigenetic immune cell counting (EICC) is not subjected to such limitations, because it uses DNA as substrate. It is based on amplification of immune cell-type specifically unmethylated gene loci as biomarkers using qPCR after bisulfite conversion (11). With all cells having identical gene copies, quantifying cell type-specific DNA methylation markers allows the deduction of target cell numbers (12). The specificity of epigenetic markers for various lymphocyte populations have been shown previously (11–14).

For prognostic EICC, this method allows to provide samples to laboratory analyses without direct patient contact, particularly since capillary blood obtained from fingerpricks may be a feasible source (12). DNA can also be retrieved minimal-invasively from nasopharyngeal swabs to provide information about local disease status. At sites of infection, the immune status may be of diagnostic interest and provide early indications of the systemic disease development.

Here, we compared immune cell counts of capillary blood dried on filter paper (dried blood spots, DBS) with venous blood from healthy donors and investigated a diagnostic value of epigenetic biomarkers along the course of COVID-19. Blood from patients reported at two different clinical sites were measured using epigenetic markers for CD3^+^, CD4^+^, CD8^+^ and regulatory T cells (Treg), total, memory and naïve B lymphocytes, natural killer (NK) cells and neutrophils. We assessed if the epigenetic markers could be used alongside COVID-19 specific qPCR analyses from nasopharyngeal swabs for reporting local immune response.

The aim of our study was to investigate whether EICC is a potential tool to bridge gaps between prognostic laboratory analyses and mandated (home) isolation providing COVID-19 prognosis. Given the feasibility to perform EICC from nasopharyngeal swabs and DBS, we assume that this approach lends itself to unsupervised home testing and possibly facilitate medical surveillance.

## Materials and methods

### Cohorts

Peripheral blood of 103 unvaccinated COVID-19 patients were collected either at Ruhr-University Bochum and University Hospital Essen in Germany (“Bochum cohort”) or at University General Hospital of Valencia, Spain (“Valencia cohort”) (**Supplementary Tables 1**, **2**). The Bochum cohort included 173 blood samples from 81 hospitalised patients and up to four time points. The median time between admission and the first visit was one day (IQR 0-4). For 75 patients the blood sample from the first visit was available. This cohort comprised of patients that were classified as cases with moderate (n = 48), severe (n = 19), critical (n = 9), or unknown symptoms (n = 5). Clinical pictures of the moderate cases were heterogeneous including asymptomatic patients as well as those with various but non-severe respiratory symptoms. The Valencian cohort included 90 blood samples from 22 patients collected at up to 10 time points upon admission to intensive care unit (ICU). The 22 Valencian patients were admitted to hospital with severe (n = 21) or critical (n = 1) symptoms (for survival, comorbidities, and treatments see **Supplementary Tables 1**–**3**). For each time point, the disease stage was assigned according to the German Robert-Koch-Institute (RKI) classification (15).

113 blood samples of self-reported healthy, of European descent, 18-71 years old pre-pandemic donors (n = 113 individuals) were obtained from a commercial supplier (in.vent Diagnostica GmbH, Germany).

For EICC in nasopharyngeal swabs, 69 samples from 23 healthy donors (median age 32, ranging from 24 to 55 years) were collected. 45 samples from COVID-19 patients (median age 74, n = 28, ranging from 35 to 91 years; **Supplementary Table 4**) were collected at University Hospitals Ruhr-University Bochum and Essen (Germany).

### Sample preparation

75 µl capillary blood was collected in a Microvette^®^ 200 tube (Sarstedt) containing EDTA and was dispensed on DBS, i.e., Whatman 903™ protein saver card. A single DBS punch (6 mm diameter) was used for comparison with matched venous blood (20 µl) collected from the same donor. DBS and their respective blood samples were lysed using 80 µl lysis solution (23.75 µl nuclease-free H_2_O [CAS-No.: 7732-18-5], 20 µl λ-DNA [37.5 ng/µl, CAS-No.: 91080-14-7], 5.25 µl proteinase K [PK, 30 mg/ml, CAS-No.: 39450-01-6], 31 µl Lysis-Binding-Buffer [LBB, Invitrogen™ Dynabeads™ SILANE Genomic DNA Kit]) for 20 min at 56°C and 1400 rpm. Peripheral whole blood (EDTA) samples (200 µl) were lysed by adding 144.7 µl LBB and 24 µl PK and incubated for 60 min at 56°C and 1400 rpm.

Nasopharyngeal swabs (FLOQSwabs, COPAN Diagnostics) were placed into a collection tube containing 0.5-2.0 ml NaCl-Solution (0.9%, CAS-No.: 7647-14-5). Genomic DNA was extracted from the resulting suspension using QIAamp Blood Midi Kit (Qiagen) according to manufacturer’s protocol. The eluate was dried at 60°C *via* concentrator (Eppendorf) and reconstituted in 75 µl nuclease-free H_2_O.

### Bisulfite conversion

75 µl of DNA solution or lysate was incubated with 135 µl ammonium bisulfite solution (65-75% [w/w], CAS-No.: 10192-30-0) and 45 µl tetrahydrofuryl alcohol (THFA, purity ≥ 98%, CAS-No.: 97-99-4) at 80°C and 900 rpm for 45 min. DNA was then (re-)purified using magnetic beads (Mag-Bind^®^ Blood & Tissue DNA HDQ 96 Kit, Omega Bio-tek).

### Cell counting by epigenetic qPCR

Cell type-specific differentially methylated regions have been identified and validated for CD3^+^, CD4^+^, CD8^+^ T cells, Treg, neutrophils, total B and NK cells (12–14). Moreover, DNA methylation markers specific for memory and naïve B cells, which were identified in a genome-wide discovery on Illumina’s Infinium MethylationEPIC BeadChip assay, were used (**Supplementary Figures 1**, **2**). These markers are based on CpGs cg18647989 and cg21855816 (Illumina ID) and are associated with genes *CBX6* (ENSG00000183741) and *C7orf50* (ENSG00000146540), respectively. Based on these markers, qPCR was performed aimed at cell counting using specifically developed qPCR systems. In brief, reactions were set up in 10 µl using the LightCycler^®^ 480 Probes Master chemistry (Roche) containing 3 µl of template DNA, 5 ng λ-DNA and target-specific primers and probes at concentrations as indicated in **Supplementary Table 5**. Amplification was performed in 384-multiwell plates using Lightcycler 480II instruments (Roche) starting with 35 min at 95°C followed by 50 cycles each at 95°C for 15 sec and 61°C for 1 min.

Relative quantification is based on measurement of cell type-specific demethylation relative to the total cell number as determined by the house-keeping gene *glyceraldehyde-3-phosphate dehydrogenase* (*GAPDH*), which has been shown to be fully demethylated in all blood cells (12). The target cell count (%) in a sample is given by the quotient of the cell type-specific (e.g., *CD3*) and *GAPDH*-specific demethylation expressed by measured demethyl-specific copy number per reaction multiplied by 100 (or 200 in case of X-linked loci, e.g., FOXP3). Copy numbers are calculated based on measured Cp values of an internal standard curve resulting from the parallel measurement of a serially diluted in silico-converted plasmid (GenScript Biotech Corp.) that contains assay target sequences for target cell types and GAPDH.

Due to present differences in assay efficiencies, the relative cell count needs to be normalized by division with an assay-specific calibration-factor. Therefore, a calibrator (plasmid containing the genomic assay target sequences) is converted, purified, and analysed in parallel with samples and the resulting target assay-specific and GAPDH-specific copy number from the calibrator is used to calculate the calibration-factor (calibration-factor = target assay-specific [copies]/GAPDH-specific [copies]). This results in an assay-specific calibration-factor used for normalisation of measured copies to GAPDH copies. The procedure was previously described with more details by Baron et al. (12).

### Flow cytometry

CD45^+^, CD19^+^, CD56^+^, CD3^+^, CD4^+^ and CD8^+^ T cells were counted using the BD Multitest 6-color TBNK kit (BD Biosciences Cat.-No. 337166, RRID: AB_2868707). 20 µl of the BD Multitest 6-color TBNK antibody were added into 5ml Trucount tubes and 50 µl whole blood (EDTA) was added. After an incubation step at 21°C for 15 min in the dark, 450 µL of pre-warmed (37°C) 1 x Red blood cell lysis buffer was added to the tubes and again incubated. Cell counts were acquired using a LSRFortessa X-20 and BD FACS DIVA software (v8.0) and data analysis was performed using FlowJo Software (v10.6).

### Statistical analysis

Both, cell counts and percentages are used to report observations of categorical variables. Median statistics are reported for continuous variables. Significant median differences between cohorts or groups were assessed by non-parametric Wilcoxon rank sum test or Fisher’s exact test. Results were considered statistically significant at a two-sided p value of less than 0.05. As this study consists of exploratory analyses only, the tests are not corrected for multiplicity. Kolmogorov-Smirnov test was used to assess whether cohort populations, for each disease stage respectively, can be assumed to be drawn from one parent population. Prognostic performance for markers for a good prognosis was tested by receiver operating characteristic (ROC) analysis, encoding good prognosis as 0 and poor prognosis as 1. For all markers, the threshold is used to classify values above as 1. Area under the curve (AUC) (95% CI) was calculated and sensitivity, specificity, accuracy, and optimal threshold were determined by Youden’s J statistic (16). Multivariate analysis was performed using a logistic regression (threshold = 0.5), predicting the outcome (poor prognosis > 0.5, good prognosis < 0.5) based on a choice of markers after univariate analysis. All analyses were performed in R (version 4.1.3.).

## Results

### Comparison of epigenetic immune cell quantification with flow cytometry

**Figure 1:**
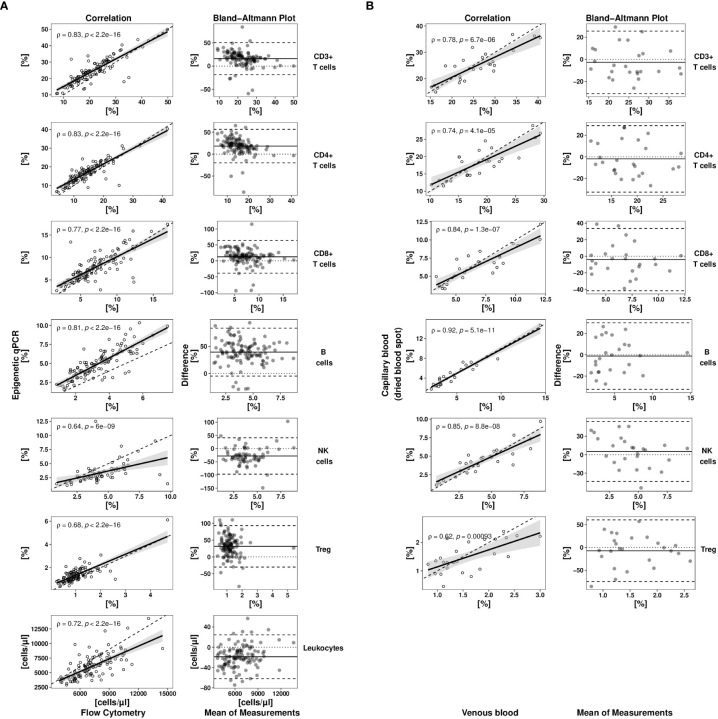
Method comparison studies for epigenetic cell quantification. **(A)** A full method comparison study of epigenetic qPCR- and flow cytometry-based cell quantification in venous whole blood samples of healthy donors (n = 113) was undertaken for different cell types. **(B)** Immune cell counts (epigenetic qPCR) in capillary blood (stored as dried blood spot (DBS)) of healthy donors (n = 25) were compared to matched venous blood samples (liquid). Each method comparison is displayed by a scatterplot (left side) and a tukey mean difference plot (right side). Scatter plot showing immune cell frequencies determined by epigenetic qPCR plotted against flow cytometrically determined relative or absolute cell numbers (dashed line: bisectrix, solid line: linear regression line (y~x) with 95% confidence interval). Spearman (rho) coefficients with corresponding p values are shown in correlation plots. Tukey mean difference plot shows difference normalized by the mean of both methods for each sample expressed as percentage (dashed lines indicate -/+ 1.96-times standard deviation of relative difference, solid line indicates systematic error (bias)). Treg, regulatory T cells; NK cells, CD56dim natural killer cells.

For method comparison between flow cytometry (the pertinent gold standard of lymphocyte quantification) and EICC, freshly drawn EDTA-whole blood samples from 113 pre-pandemic healthy donors were analysed (**Figure 1A**). Cell counting was performed with standard flow cytometry as well as epigenetic markers for CD3^+^, CD4^+^, CD8^+^ T cells, Treg, CD19^+^ B, and CD56^+^ NK cells. Comparisons indicated strong association between both methods (Spearman rho between 0.64 and 0.83) with method dependent biases ranging from 12.6% for CD8^+^ T cells to 39.6% for B cells. Limits of agreement (LoA) were comparably high when combinations of different flow cytometric markers were used, such as CD3^-^ CD16^low^ CD56^+^ for NK cells (35.2%) or CD4^+^CD25^+^CD127^-^FOXP3^+^ for regulatory T cells (31.4%) than for more basic markers, such as CD3^+^ or CD3^+^CD4^+^ (LoA_CD3_ = 17.7%, LoA_CD4_ = 19.6%) (**Table 1**). Additionally, EICC showed substantial equivalence when comparing data from peripheral blood with capillary blood on filter paper (DBS) for all tested markers (LoA: 14.4 to 35.2%, Bias: -1.2 to -28.0%) (**Figure 1B** and **Table 1**).

**Table 1:** Method comparisons.

Cell type	qPCR versus flow cytometry			Capillary versus venous blood		
	Spearman`s rho (p-value)	Bias (95% CI) [%]	Limit of Agreement (95% CI) [%]	Spearman`s rho (p-value)	Bias (95% CI) [%]	Limit of Agreement (95% CI) [%]
T cells [%]	0.83 (<0.0001)	16.08 (14.42, 17.74)	17.66 (15.31, 20.00)	0.78 (< 0.0001)	-2.65 (-5.53, 0.23)	14.40 (10.33, 18.47)
T helper cells [%]	0.83 (<0.0001)	18.39 (16.56, 20.23)	19.56 (16.96, 22.16)	0.74 (< 0.0001)	-1.86 (-5.01, 1.29)	15.74 (11.29, 20,19)
Cytotoxic T cells [%]	0.77 (<0.0001)	12.57 (10.10, 15.04)	26.27 (22.77, 29.76)	0.84 (< 0.0001)	-4.05 (-7.90, -0.21)	19.20 (13.77, 24.63)
B cells [%]	0.81 (<0.0001)	39.55 (37.43, 41.68)	22.61 (19.61, 25.62)	0.92 (< 0.0001)	-1.18 (-4.36, 2.01)	15.92 (11.42, 20.43)
NK cells [%]	0.64 (<0.0001)	-28.04 (-32.37, -23.71)	35.16 (29.04, 41.28)	0.64 (< 0.0001)	-28.04 (-32.37, -23.71)	35.16 (29.04, 41.28)
Regulatory T cells [%]	0.68 (<0.0001)	31.44 (28.47, 34.41)	31.41 (27.21, 35.61)	0.62 (0.0009)	-7.09 (-13.99, -0.19)	34.50 (24.74, 44.26)
Leukocytes [cells/µl]	0.72 (<0.0001)	-18.75 (-20.83, -16.68)	22.06 (19.12, 24.99)	NA	NA	NA

All indicated cell types were measured with pertinent epigenetic and flow cytometry markers using venous blood (left column) or only with epigenetic markers using venous and capillary blood samples (right column). Spearman correlation coefficient indicates rank associations between measurements of the same sample. Bias is determined as the mean relative measurement differences between methods. Limit of agreement describes the standard deviation of the relative measurement differences.

### Lymphocyte counts in COVID-19 patients at initial hospital admission

**Figure 2:**
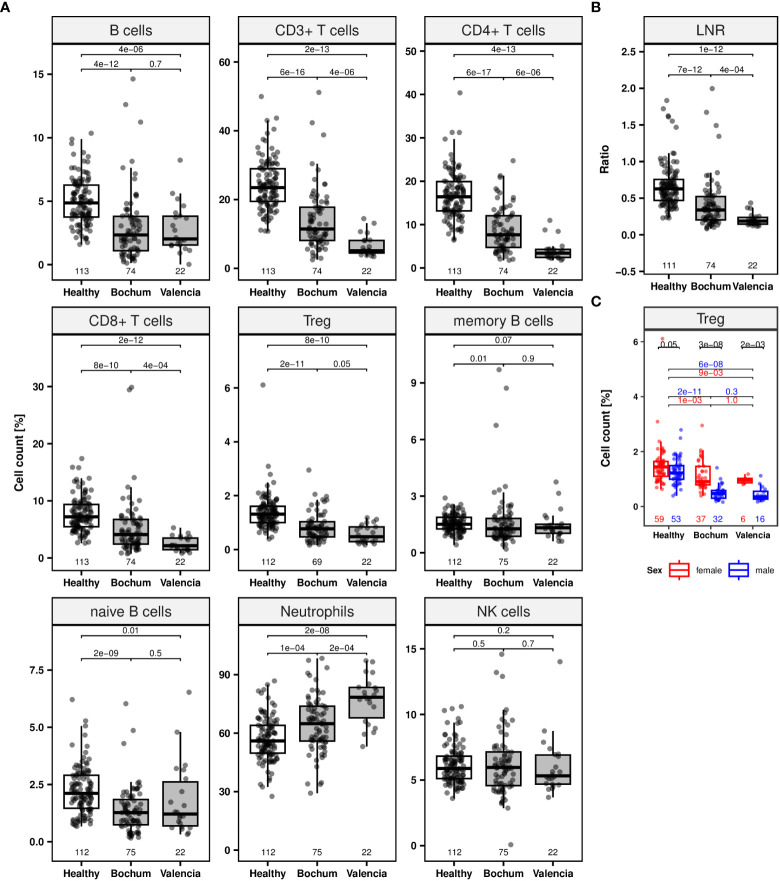
Lymphocyte populations of COVID-19 patients at initial hospital admission. Jittered scatters indicate actual cell count for a single sample at the first visit is defined as timepoint at initial admission. Boxes display the interquartile range and different coloured boxes indicate healthy (white box, n = 113) or COVID-19 patient cohorts from two clinical sites (grey boxes, Bochum, n = 75; Valencia, n = 22). Whiskers extend maximally 1.5 times the interquartile range from the upper/lower end of the box. Observations farther than that are considered outliers. All p values relate to the Wilcoxon rank sum test for median differences and are displayed above the respective boxplots. **(A)** Boxplots for lymphocyte subpopulations (CD19^+^ B, CD3^+^, CD4^+^, CD8^+^, regulatory T (Treg), memory, naïve B cells, neutrophils and CD56dim natural killer [NK] cells). **(B)** Boxplot of lymphocyte-to-neutrophil ratio (LNR). **(C)** Boxplots for Treg counts separated by sex (colors: female = red, male = blue).

Using markers for neutrophils as well as naïve and memory B cells and the markers described above for CD3^+^, CD4^+^, CD8^+^ T cells, Treg, CD19^+^ B, and CD56^+^ NK cells, whole blood samples from COVID-19 patients (time point 1; Bochum with n = 75 and Valencia with n = 22) were epigenetically quantified and compared to samples from the healthy subjects (**Figure 2**, **Supplementary Table 6**).

Both patient cohorts show significantly lower median frequencies (%) of CD3^+^, CD4^+^, CD8^+^, and Treg at the first blood draw after hospitalization (Wilcoxon rank sum test: p < 0.0001) (**Figure 2A**). Moreover, significant deficiencies were demonstrated for total and naïve B cells for both cohorts (p < 0.05) as well as for memory B cells for the Bochum cohort (p = 0.01). No significant NK cell differences between patients and healthy subjects were found. An increase of the median neutrophil count was observed in whole blood of both patient cohorts (p < 0.0005). All comparisons of patient cohorts were against the healthy donor cohort. The median ratio of lymphocytes-to-neutrophils (LNR) was lower in patients compared to healthy subjects (p < 0.0001) (**Figure 2B**). Significantly lower median CD3^+^, CD4^+^, CD8^+^ T lymphocytes counts and LNR as well as a higher neutrophil count were observed in the Valencian cohort, when compared with the Bochum cohort (p < 0.0005). This finding is supported by Kolmogorov-Smirnov statistics which indicated significant differences (p < 0.05) for various cell counts prohibiting joint data analysis for both cohorts (**Supplementary Table 7**).

An interesting observation was made for Treg. In healthy subjects, a small non-significant (p = 0.05) difference in the number of Treg was observed between females (1.4% [IQR 1.1-1.6], n = 59) and males (1.2% [IQR 1.0-1.5], n = 53) (**Figure 2C**; **Supplementary Table 6**). For both patient cohorts independently, this difference was dramatically increased (p ≤ 0.002). Whereas the median Treg count in females were at 0.9% (IQR 0.8-1.5, n = 37, Bochum cohort) and 1.0% (IQR 0.9-1.0, n = 6, Valencian cohort) male subjects lost the majority of Treg, with median cell levels of 0.5% and less.

### Prognosis of disease course

The counts of the various immune cell populations were assessed for a potential predictive value. Patients were grouped into two outcome classes based on the clinical status at one time point relative to their clinical performance at the next reported visit. Patients were assigned as having a “poor prognosis” when the disease status turned from moderate to either severe or critical and when the status turned from severe to critical. Patients were assigned as having a “good prognosis” when the clinical status was initially severe or critical and improved to moderate (from severe) or severe or moderate (from critical). Patients were also assigned to “good prognosis” when the clinical status remained moderate.

**Figure 3:**
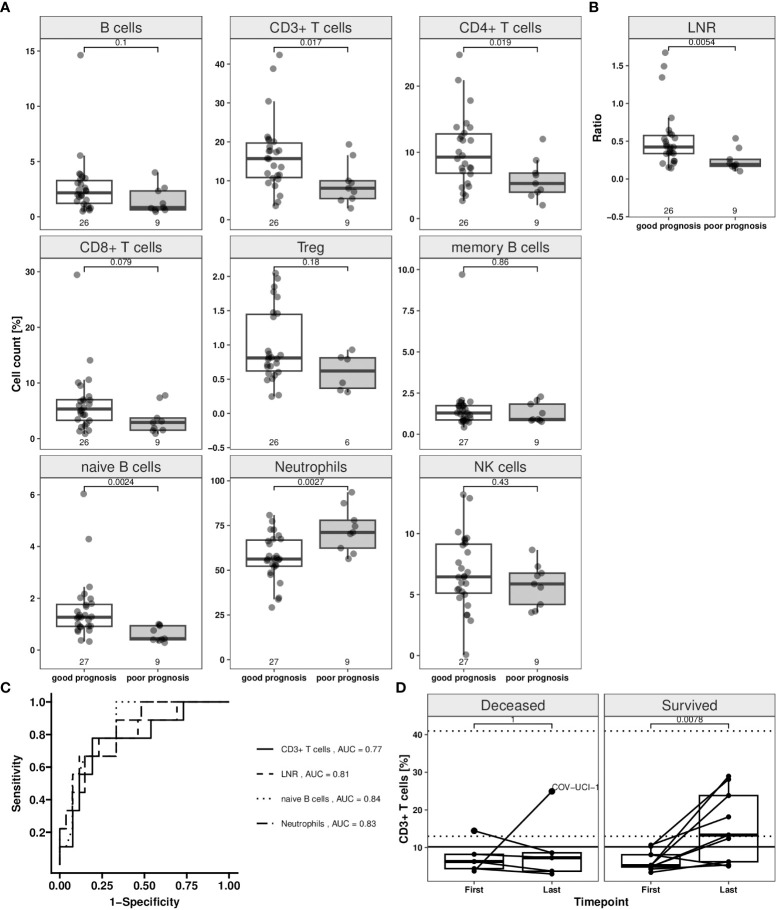
Immune cell counts depending on disease status switch in COVID-19 patients in whole blood samples. The disease status was assessed in accordance with RKI classification of hospitalized COVID-19 patients. The “good prognosis” group consists of patients (n = 27) which showed an improvement until second available time point compared to first time point. Improvement was assumed when a change from severe to moderate and persistent moderate grade was found. Patients with a change from moderate or severe to critical, moderate to severe grade were classified as “poor prognosis” group (n = 9). Patients were represented in both groups, when showing status change from second to third time point. Epigenetic data for the first and second time point (when status changed from second to third time point) are illustrated in the plots (white box: good prognosis; grey box: poor prognosis). Jittered scatter indicates actual cell count for a single sample. **(A)** Boxplots for T and B lymphocyte subpopulations (CD19^+^ B, CD3^+^, CD4^+^, CD8^+^, regulatory T (Treg), memory, naïve B cells), neutrophils and CD56dim natural killer [NK] cells. **(B)** Boxplot for lymphocyte-to-neutrophil ratio (LNR). **(C)** Prognostic performance for the markers CD3^+^ T cells, LNR, naïve B cells and neutrophils for a good prognosis was assessed by “receiver operating characteristic” (ROC) analysis, encoding good prognosis as 0 and poor prognosis as 1. Calculated area under the curve (AUC) for the four markers are shown in the plot. **(D)** CD3^+^ T cell course of Valencia cohort from first and last time point. Dynamic changes of CD3^+^ T lymphocytes in COVID-19 patients admitted into hospital. Relative numbers of CD3^+^ T lymphocytes are analysed at first and last available time point after hospital admission. Only patients with more than two time points were included in this analysis. Solid horizontal line shows the CD3^+^ T cell threshold of ≥ 10.2% that marks the count that was defined as recovery limit. Each patient trajectory is illustrated by a line between two time points (deceased: n = 5, survived: n = 9). Dotted horizontal lines mark the normal “healthy” CD3^+^ T cell range (95% CI: 12.99–40.97%). One patient (labelled with “COV-UCI-1”) was not following the pattern. Boxes display the interquartile range. Whiskers extend maximally 1.5 times the interquartile range from the upper/lower end of the box. Observations farther than that are considered outliers. All p values relate to the Wilcoxon rank sum test for median differences and are displayed above the respective boxplots.

Relative median cell counts at the first time point for patients with poor (n = 9) and for those with good prognosis (n = 27) were compared (**Figure 3A**; **Supplementary Table 8**). CD3^+^, CD4^+^ T and naïve B cells were significantly lower for patients with poor, compared to those with a good prognosis (Wilcoxon rank sum test: p < 0.05). Median Treg, NK, overall CD19^+^, and memory B cell counts did not differ significantly between both groups. The median neutrophil count for patients with a poor prognosis measured significantly higher (p = 0.0027) than in those with good prognosis. The LNR was significantly lower in patients with poor compared to those with a good prognosis (p = 0.0054) (**Figure 3B**).

Next, we assessed CD3^+^ T, naïve B cells, neutrophils and LNR with respect to their power to predict the clinical disease course applying ROC analyses (**Figure 3C**). For CD3^+^ T cells, an area under the curve (AUC) of 0.77 (95% CI: 0.59–0.96) was calculated (**Supplementary Table 9**). The best separation point corresponded to a threshold level of 10.2% CD3^+^ T cells at which 78% of the cases with poor prognosis were detected correctly, with a false negative rate of 19%. The AUC for the LNR was at 0.81 (95% CI: 0.63–0.98) and correctly detected 67% of patients with poor prognosis. A 12% false negative rate was found, with a calculated threshold level of 0.21. CD3+ T, naïve B cells, and neutrophils were used in a multivariate logistic regression, creating a predictor for the outcome. The multivariate model resulted in a sensitivity of 88% and specificity of 67% (**Supplementary Table 9**).

### T cell recovery is associated with patient survival

Patients admitted to ICU had low T, B lymphocyte and high neutrophil counts suggestive for a severe disease course. To predict survival, these patients were then separated in those that deceased and those that survived and respective CD3^+^ T cell counts of both groups at first and last time point were compared. At the first time point (ranging from -1 to 2 days around the day of admission) no difference in the CD3^+^ T cell count was found between patients that deceased and those that were discharged (6.3% [IQR 4.4–8.2], n = 5 and 5.2% [IQR 4.8–8.1], n = 9) (**Figure 3D**). The best factor for patient survival was clearly recovery of CD3^+^ T cell count, whereas for those with critical and eventually deadly outcome, no such recovery was observed. Survivors were characterized by a significant increase of CD3^+^ T cell counts compared to the first time point (13.3% [IQR 6.2–23.8], p = 0.0078). Their cell counts corresponded to those of healthy donors (95% CI: 12.99–40.97%). The CD3^+^ T cell count was indicative for recovery. Among survivors 67% of cases showed a cell count above the calculated threshold of 10.2% for a good prognosis after a median of 12 days (IQR 11-23, ranging from 9 to 36). 80% of patients that succumbed to the disease showed no reconstitution of CD3^+^ T cells (**Figure 3D**).

### Detection of immune cells in nasopharyngeal swabs

**Figure 4:**
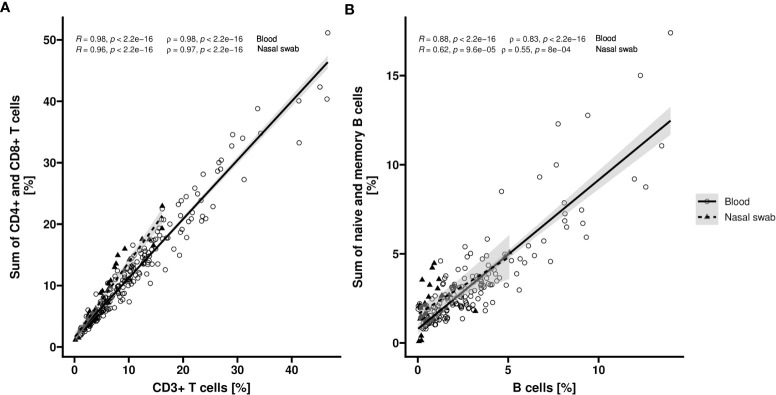
Correlation of T or B cell populations in blood and nasopharyngeal swabs. Relative counts (%) of CD3^+^, CD4^+^ and CD8^+^ cells respective overall, memory and naïve B cells are determined by epigenetic qPCR for whole blood (Blood) and nasopharyngeal swabs (Nasal swab) from COVID-19 patients or healthy volunteers. Scatter plots show the correlation of CD3^+^ T cell count and sum of CD4^+^ and CD8^+^ T cell count **(A)** (n = 213 [Blood] or n = 40 [Nasal swab]) or total B cell count and sum of naïve and memory B cell count **(B)** (n = 166 [Blood] or n = 34 [Nasal swab]). Datapoints for blood samples are indicated by circles (solid line) and for nasopharyngeal samples by triangles (dashed line). Corresponding regression lines (y~x) are shown as solid lines inclusive 95% confidence interval (grey area). Pearson (R) and spearman (rho) coefficients with corresponding p values are shown in plot.

To assess the cell type specificity of the epigenetic markers for CD3^+^, CD4^+^, and CD8^+^ T cells as well as total B, naïve, and memory B cells in nasal swabs we performed a joint data analysis of qPCR data of healthy donors and COVID-19 patients in comparison to blood. In both tissues, CD4^+^ and CD8^+^ T subpopulations showed a clear linear association with the number of overall CD3^+^ T cells (blood: R = 0.98, rho = 0.98 [p < 0.0001], n = 213; nasal swab: R = 0.96, rho = 0.97 [p < 0.0001], n = 40) (**Figure 4A**). In blood, memory and naïve B cell frequencies correlated with total B cell frequency (R = 0.88, rho = 0.83 [p < 0.0001], n = 166) (**Figure 4B**). For nasal swabs, a moderate correlation between numbers of analysed B cell subpopulations and total B cells was observed (R = 0.62, rho = 0.55 [p < 0.001], n = 34).

**Figure 5:**
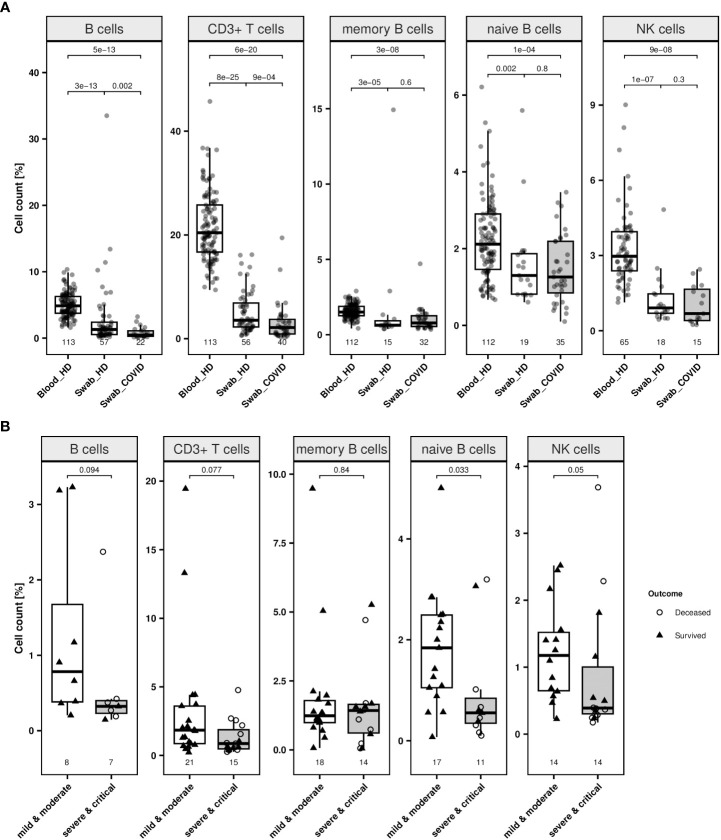
Lymphocytes in swabs from healthy donors and COVID-19 patients. **(A)** Relative quantification of lymphocyte populations in nasopharyngeal swab samples from healthy donors (labelled as “Swab_HD”) and COVID-19 patients at first available time point (labelled as “Swab_COVID”) compared to blood cell count of healthy individuals (labelled as “Blood_HD”) were determined by epigenetic qPCR. Investigated cell types are CD19^+^ B cells, CD3^+^ T cells, memory and naïve B cells and CD56dim natural killer (NK) cells. **(B)** Relative quantification of lymphocyte populations in nasopharyngeal swab samples from COVID-19 patients (all time points) with mild or moderate symptoms (white box) compared patients with severe or critical illness (grey box). Outcome respective status at discharge of the patients is indicated by different point shapes (deceased patient: circle, survived patient: triangle). Data are presented as boxplot and compared by Wilcoxon rank sum test. Boxes display the interquartile range. Whiskers extend maximally 1.5 times the interquartile range from the upper/lower end of the box. Observations farther than that are considered outliers. All p values relate to the Wilcoxon rank sum test for median differences and are displayed above the respective boxplots.

Next, we analysed 69 samples from 23 healthy donors and performed epigenetic analyses targeting CD3^+^ T, total, memory, and naïve B as well as NK cells, to assess feasibility of EICC in nasopharyngeal swabs. Compared to blood from healthy donors, the median counts of all investigated cell types measured significantly lower in swabs from healthy volunteers (Wilcoxon rank sum test: p ≤ 0.002) (**Figure 5A**). The median CD3^+^ T, overall, memory and naïve B, and NK cell count in swabs from healthy volunteers were at 3.6% (IQR 2.3–6.9), 1.3% (0.5–2.4), 0.6% (0.6–0.9), 1.3% (0.8–1.9), and 0.9% (0.7–1.5), respectively (**Supplementary Table 10)**.

Moreover, swabs from COVID-19 patients, irrespective of the clinical progression of the patient, showed a significantly lower CD3^+^ T and B cell count (p ≤ 0.002) (**Figure 5A**). When associating immune cell counts in swabs to different clinical stages and survival, patients experiencing moderate symptoms (n = 27) had a significantly higher median percentage of naïve B (p = 0.033) than patients with severe or critical disease (n = 17), respectively (**Figure 5B**; **Supplementary Table 10**). Other cell types did not differ significantly between milder and more severe cases.

## Discussion

EICC is feasible with DNA samples derived from tissues and body fluids including fresh, frozen, and dried blood. Substantial equivalence with pertinent flow cytometric analyses was shown using peripheral blood (**Table 1**), and independently confirms and expands previous findings (12). For EICC, equivalence was also found for venous blood and capillary blood collected as DBS.

Compared to blood, significantly lower immune cell frequencies were observed in nasopharyngeal swabs, which is consistent with a low infiltration of lymphatic cells in that tissue (17). In blood and nasopharynx, epigenetically detected CD4^+^ and CD8^+^ T cells correlated well with the number of total CD3^+^ T cells and memory and naïve B cells correspond moderately well with overall B cells. This suggests an equivalent cell type specificity of the epigenetic markers in both tissue types. When comparing cell counts in swabs and matched peripheral blood samples, we found no reliable correlation (data not shown).

To investigate the predictive value of the epigenetic approach with respect to the disease outcome after SARS-CoV-2 infections, immune cell levels were monitored. Patients have significantly reduced counts of T and B cell populations compared to healthy donors. This is in agreement with previous findings (18–20). Moreover, the multivariate analysis indicated that counts of T cell subpopulations as well as naïve B cells, neutrophils and the LNR at first time point were predictive for the clinical stage at the consecutive clinical patient assessments (**Supplementary Table 9**), supporting previous reports (18, 21, 22). This finding suggests that these markers precede clinical manifestation of COVID-19 at early stages serving as early indicators for disease course. The same was not observed for overall or memory B cells or NK cells, confirming previous findings (3, 23). This points towards cell type specific effects rather than a general granulocytosis, which would lead to an equal reduction of all lymphocyte populations (24). We also observed that T cell lymphopenia and neutrophilia correlated with increasing disease severity (**Supplementary Table 11**).

Any effect of treatment (Remdesivir-treated, Tocilizumab-treated) or present comorbidities like arterial hypertonia (38% of all patients) or diabetes mellitus type 2 (25%) on the immune cell counts of each patient cohort, for instance, was not observed (data not shown). Jointly, these observations suggest that EICC in blood represent reliable stage-associated laboratory parameters.

The advanced lymphopenia within the Valencian cohort at admission might be explained by the fact that almost all patients were severely ill and in intensive care, compared to the German cohort that included many non-severe cases in stationary care. However, within the highly lymphopenic Valencian cohort, we observed T cell recovery until end of treatment in patients who survived. T lymphocyte counts in terminal patients did not recover during observation (**Figure 3D**), confirming reported data (10, 25).

Furthermore, our data indicate that DBS can be used to predict disease course. As logistics for DBS are simple, patients in home isolation may be able to send specimens to laboratories. The following analyses are feasible in approx. 6 hours and would allow for timely monitoring. While such strategy may lack complete medical surveillance, it allows for objective immune monitoring of patients in home isolation. Nasopharyngeal swabs are used as common source for detection of SARS-COV-2 and are usually available upon COVID-19 diagnosis. Significant reduction of T and B cells for patients was observed when compared to healthy donors. Notably, naïve B cell levels were predictive in blood and swabs. A role of the naïve immune system is in line with the observed age dependency of COVID-19 (26, 27).

In addition to corroborating the finding that high T lymphocyte and low neutrophil counts determine better outcome, our data show one possible reason for well-established sex-dependency of COVID-19 outcome (28–30). Treg appear to be disappearing almost entirely from the periphery of male patients. Contrary to that, females retain a Treg count of approx. 1.0%.

Cohort size was a limitation of this study. Unexpected differences in the source populations from Valencia and Bochum barred the aggregation of samples to one larger cohort. Also, the current experiment does not provide an immediate diagnostic use case, but immune monitoring appears feasible from tissue samples. By design of ethical consent, direct testing of home isolated patients was not allowed in the study. Therefore, the feasibility of this study is limited to the technical aspects. An actual proof of benefit of this method for patients in home-isolation must follow this study. Validation studies using larger cohorts including multicentric studies from different regions and countries and DBS, including mildly ill out-patients, are essential to confirm the full prognostic potential for COVID-19 patients.

Overall, we demonstrated the applicability of EICC in venous and capillary blood and nasopharyngeal swabs for predicting COVID-19 disease progression and emphasised to be aware of the sex-related differences in regulatory T cell counts when assessing individual disease course. Furthermore, given the feasibility to perform EICC from nasopharyngeal swabs and DBS, this approach lends itself to unsupervised home testing and possibly facilitate medical surveillance. The concept shown here may be transferred to other clinical applications, such as newborn screening for primary immunodeficiencies using DBS (31) or determination of immune cell infiltration in solid tumor tissues (32), where flow cytometry in particular is not suitable or reaches its limits, as well as to situations where patients suffer from chronic inflammatory pathologies like rheumatoid arthritis or systemic lupus erythematosus (33, 34).

## Data availability statement

The datasets presented in this study can be found in online repositories. The names of the repository/repositories and accession number(s) can be found below: https://doi.org/10.6084/m9.figshare.21710237.v1.

## Ethics statement

The studies involving human participants were reviewed and approved by ethical committees of the Ruhr-University Bochum (20-6886), University Hospital Essen (20-9214-BO) and of University General Hospital of Valencia (65/2020). The patients/participants provided their written informed consent to participate in this study.

## Author contributions

SO conceived the study, developed the methodology and was responsible for conceptualization and project administration. BS and KS did the formal analyses. BS, JJ, AR, and LL did the investigation. MR, LA, JH, and NB were responsible for resources. BS and SO supervised the study. BS and KS validated the data. BS and KS were responsible for data visualization. BS and SO wrote the original draft. SO had access to the underlying data. All authors contributed to the article and approved the submitted version.
