# Corrigendum: Epigenetic immune monitoring for COVID-19 disease course prognosis

**DOI:** 10.3389/fimmu.2024.1451571

**Published:** 2024-06-27

**Authors:** Björn Samans, Marta Rosselló Chornet, Araceli Rosselló Chornet, Janine Jung, Konstantin Schildknecht, Laura Lozza, Lourdes Alos Zaragoza, Javier Hernández Laforet, Nina Babel, Sven Olek

**Affiliations:** ^1^ Ivana Türbachova Laboratory for Epigenetics, Epiontis, Precision for Medicine GmbH, Berlin, Germany; ^2^ Charité – Universitätsmedizin Berlin, corporate member of Freie Universität Berlin and Humboldt Universität zu Berlin, Berlin, Germany; ^3^ Department of Anesthesiology and Resuscitation, Consortium General University Hospital of Valencia, Valencia, Spain; ^4^ Center for Translational Medicine, Medical Clinic 1, Marien Hospital Herne, University Hospitals of the Ruhr-University of Bochum, Herne, Germany

**Keywords:** COVID-19, SARS-CoV-2, epigenetic qPCR, immune monitoring, lymphopenia, disease prognosis

In the published article, there was an error regarding the affiliations for Björn Samans. As well as having affiliation 1, they should also have affiliation “Charité – Universitätsmedizin Berlin, corporate member of Freie Universität Berlin and Humboldt Universität zu Berlin, Berlin, Germany”.

The authors apologize for this error and state that this does not change the scientific conclusions of the article in any way. The original article has been updated.

