# Supplementary material for: Epigenetic immune monitoring for COVID-19 disease course prognosis
Source: Front Immunol. 2023 Mar 14;14:1107900. doi: 10.3389/fimmu.2023.1107900 (PMC10043382; doi:10.3389/fimmu.2023.1107900)
Supplement: Supplementary file 1 [file DataSheet_1.docx]

***Supplementary Material***


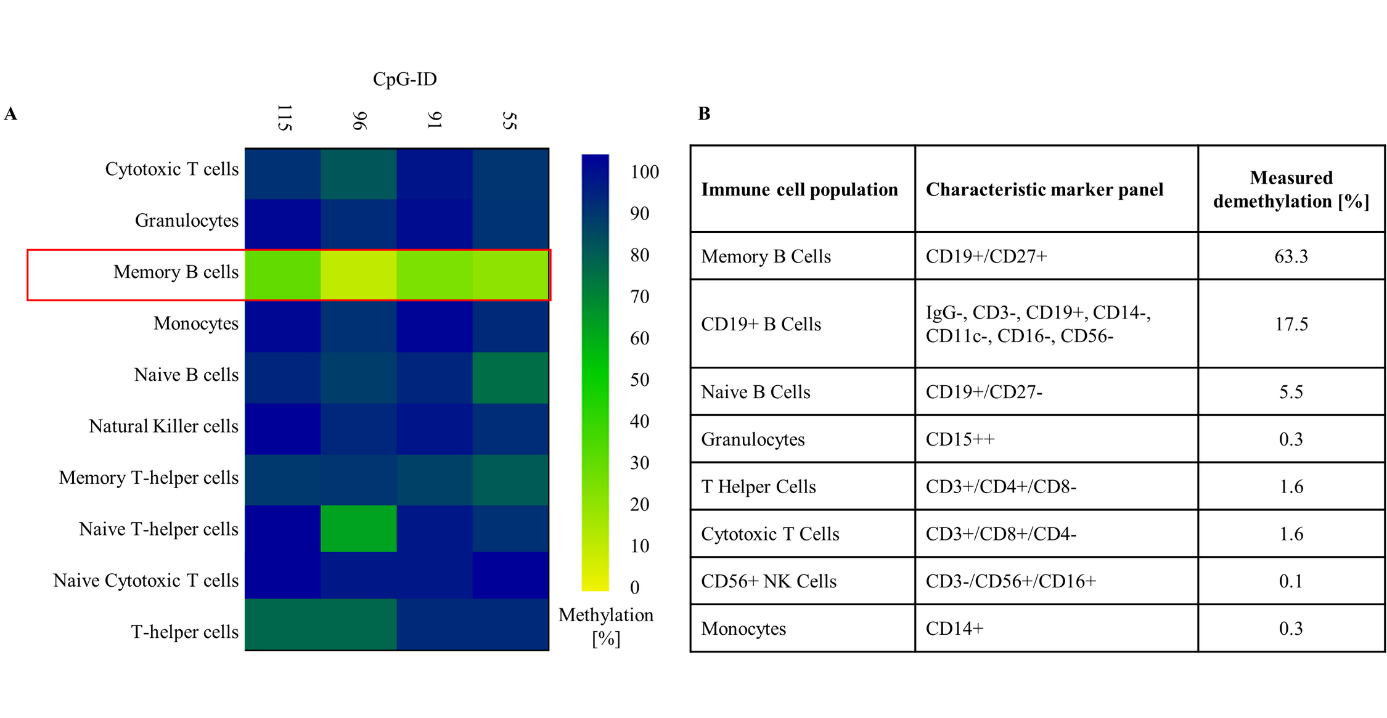


1. **Supplementary Figure 1. Epigenetic qPCR assay for the detection of memory B cells.** (**A**) Illustration of a differentially methylated region (DMR) within *CBX6* (Chromobox protein homolog 6) as a potential marker candidate for the identification of memory B cells. On isolated immune cell populations (as indicated on the Y-axis), CpG methylation values were determined by quantitative bisulfite sequencing as described recently [28]. CpG sites are identified according to their position relative to the amplicon sequence (X-axis) and measured methylation values (in %) are color-coded according to bar in the middle. Blue corresponds to full methylation (100%) and yellow indicates complete demethylation (0%). (**B)** Results from the qPCR-based quantification of demethylation (in %) of the marker region within *CBX6* shown in (**A)** on isolated immune cell preparations as indicated.


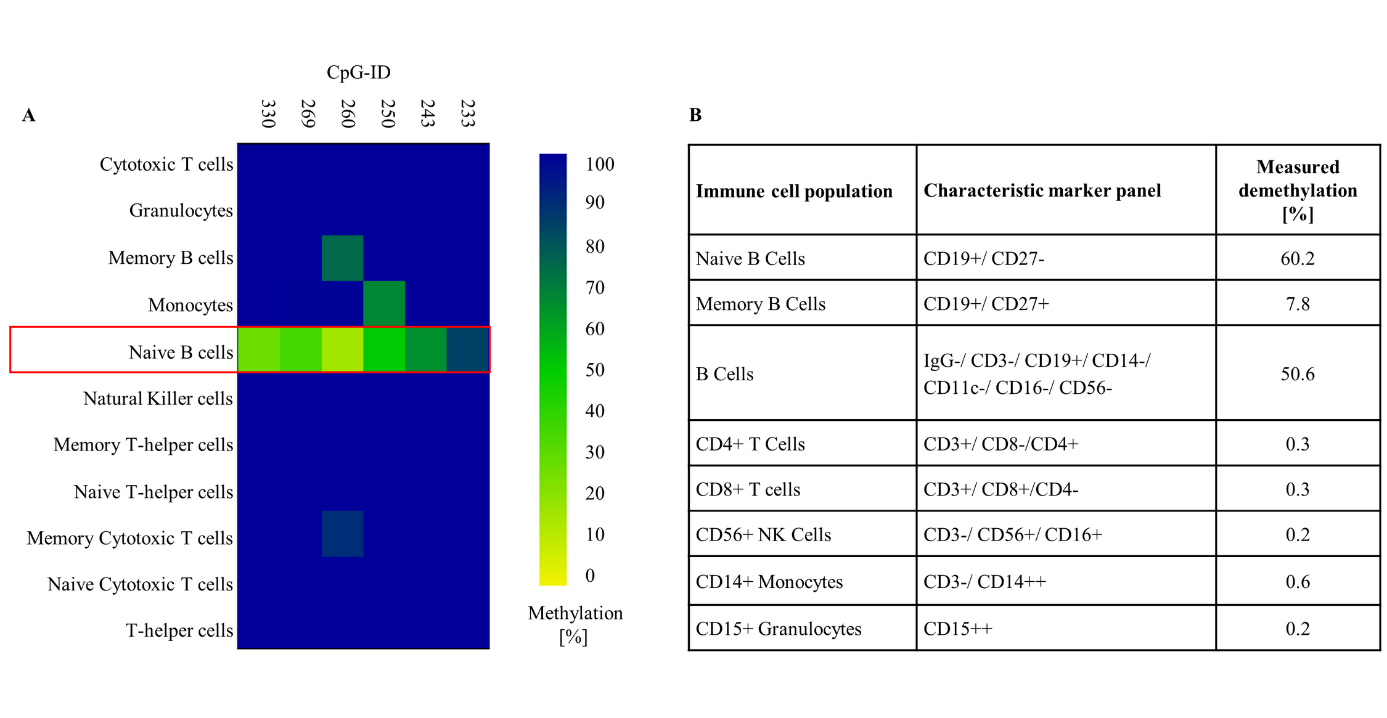


1. **Supplementary Figure 2. Epigenetic qPCR assay for the detection of naive B cells.** (**A**) Illustration of a differentially methylated region (DMR) within *C7orf50* (chromosome 7 open reading frame 50) as a potential marker candidate for the identification of naïve B cells. On isolated immune cell populations (as indicated on the Y-axis), CpG methylation values were determined by quantitative bisulfite sequencing as described recently [28]. CpG sites are identified according to their position relative to the amplicon sequence (X-axis) and measured methylation values (in %) are color-coded according to bar in the middle. Blue corresponds to full methylation (100%) and yellow indicates complete demethylation (0%). (**B)** Results from the qPCR-based quantification of demethylation (in %) of the marker region within *C7orf50* shown in **(A)** on isolated immune cell preparations as indicated.

**Supplementary Table 1. Patient characteristics for Valencia cohort.**

|  |  | **Outcome** | |  |
| --- | --- | --- | --- | --- |
| **Characteristic** | **Overall, N = 22** | **Deceased, N = 6** | **Survived, N = 16** | **p value^2^** |
| **Sex** |  |  |  | 0.6 |
| F | 6 / 22 (27%) | 1 / 6 (17%) | 5 / 16 (31%) |  |
| M | 16 / 22 (73%) | 5 / 6 (83%) | 11 / 16 (69%) |  |
| **Age** |  |  |  | 0.6 |
| Median (IQR) | 58.0 (51.5, 72.8) | 62.5 (53.0, 73.5) | 58.0 (43.0, 67.0) |  |
| Range | 24 - 78 | 51 - 77 | 24 - 78 |  |
| **Grade** |  |  |  | 0.3 |
| Severe | 21 / 22 (95%) | 5 / 6 (83%) | 16 / 16 (100%) |  |
| Critical | 1 / 22 (4.5%) | 1 / 6 (17%) | 0 / 16 (0%) |  |
| **Remdesivir treated** | 4 / 22 (18%) | 1 / 6 (17%) | 3 / 16 (19%) | >0.9 |
| **Tocilizumab treated** | 20 / 22 (91%) | 6 / 6 (100%) | 14 / 16 (88%) | >0.9 |
| **Days between admission and collection** |  |  |  | 0.4 |
| Median (IQR) | 0.0 (0.0, 0.0) | 0.0 (0.0, 0.0) | 0.0 (0.0, 0.0) |  |
| Range | -1 - 2 | -1 - 0 | -1 - 2 |  |
| **Days between positive PCR and admission** |  |  |  | 0.2 |
| Median (IQR) | 5.5 (4.0, 9.0) | 8.0 (7.2, 8.8) | 5.0 (1.8, 9.5) |  |
| Range | 1 - 72 | 4 - 19 | 1- 72 |  |
| **Days between positive PCR and first visit** |  |  |  | 0.2 |
| Median (IQR) | 5.5 (4.0, 9.0) | 8.0 (6.5, 8.8) | 5.0 (2.0, 9.5) |  |
| Range | 1 - 71 | 4 - 19 | 1 - 71 |  |
| **Observational period (days)** |  |  |  | 0.6 |
| Median (IQR) | 10.5 (5.2, 15.8) | 10.5 (7.8, 18.5) | 10.0 (5.0, 13.2) |  |
| Range | 3 - 36 | 6 - 22 | 3 - 36 |  |
| **Days between visits** |  |  |  | 0.8 |
| Median (IQR) | 3.8 (3.4, 5.0) | 3.9 (3.6, 4.0) | 3.7 (3.0, 5.0) |  |
| Range | 1 - 6 | 3 - 6 | 1 - 6 |  |

^1^ n / N (%)
^2^ Fisher exact test; Wilcoxon rank sum test

**Supplementary Table 2. Patient characteristics for Bochum cohort.**

|  |  | **Sex** | |  |
| --- | --- | --- | --- | --- |
| **Characteristic** | **Overall, N = 81** | **Female, N = 43** | **Male, N = 38** | **p value^2^** |
| **Age** |  |  |  | 0.11 |
| Median (IQR) | 69.0 (55.0, 81.0) | 65.0 (50.0, 81.0) | 75.5 (57.8, 80.8) |  |
| Range | 29 - 91 | 29 - 91 | 41 - 91 |  |
| **Grade** |  |  |  | 0.2 |
| Asymptomatic | 4 / 76 (5.3%) | 2 / 40 (5%) | 2 / 36 (5.6%) |  |
| Mild | 7 / 76 (9.2%) | 6 / 40 (15%) | 1 / 36 (2.8%) |  |
| Moderate | 37 / 76 (58.7%) | 20 / 40 (50%) | 17 / 36 (47.2%) |  |
| Severe | 19 / 76 (25.3%) | 10 / 40 (25%) | 9 / 36 (24.8%) |  |
| Critical | 9 / 76 (12.3%) | 2 / 40 (5%) | 7 / 36 (19.8%) |  |
| Unknown | 5 | 3 | 2 |  |
| **Outcome** |  |  |  | 0.3 |
| Deceased | 9 / 69 (13%) | 3 / 36 (8.3%) | 6 / 33 (18%) |  |
| Survived | 60 / 69 (87%) | 33 / 36 (92%) | 27 / 33 (82%) |  |
| Unknown | 12 | 7 | 5 |  |
| **Remdesivir treated** | 7 / 26 (27%) | 3 / 14 (21%) | 4 / 12 (33%) | 0.7 |
| Unknown | 55 | 7 | 5 |  |
| **Other therapies** |  |  |  | 0.7 |
| Apixaban | 19 / 25 (76%) | 10 / 14 (71%) | 9 / 11 (82%) |  |
| Clecane | 6 / 25 (24%) | 4 / 14 (29%) | 2 / 11 (18%) |  |
| Unknown | 56 | 29 | 26 |  |
| **Days between admission and collection** |  |  |  | 0.9 |
| Median (IQR) | 1.0 (0.0, 4.0) | 1.0 (0.0, 2.8) | 1.5 (0.0, 4.0) |  |
| Range | 0 - 50 | 0 - 50 | 0 - 19 |  |
| Unknown | 1 | 1 | 0 |  |
| **Days between positive PCR and admission** | |  |  | 0.8 |
| Median (IQR) | 0.0 (-1.0, 0.5) | 0.0 (-1.0, 0.5) | 0.0 (-1.0, 0.2) |  |
| Range | -49 - 11 | -49 - 11 | -14 - 9 |  |
| Unknown | 30 | 16 | 14 |  |
| **Days between positive PCR and first visit** |  |  |  | 0.034 |
| Median (IQR) | 2.0 (1.0, 6.0) | 1.0 (1.0, 3.5) | 2.5 (2.0, 9.2) |  |
| Range | 0 - 37 | 0 - 37 | 0 - 15 |  |
| Unknown | 30 | 16 | 14 |  |
| **Observational period (days)** |  |  |  | 0.6 |
| Median (IQR) | 4.5 (1.0, 8.0) | 5.5 (1.0, 8.0) | 4.0 (1.0, 7.8) |  |
| Range | 1 - 30 | 1 - 30 | 1 - 29 |  |
| Unknown | 1 | 1 | 0 |  |
| **Days between Visits** |  |  |  | 0.7 |
| Median (IQR) | 3.5 (3.0, 5.0) | 3.5 (3.0, 4.1) | 3.8 (3.0, 5.0) |  |
| Range | 2 - 18 | 2 - 14 | 2 - 8 |  |
| Unknown | 29 | 15 | 14 |  |

^1^ n / N (%)

^2^Fisher exact test; Wilcoxon rank sum test

**Supplementary Table 3. Comorbidities for Bochum cohort.**

| **Underlying disease** | **N = 81^1^** |
| --- | --- |
| Alzheimer's disease | 1 (1.2%) |
| Amyotrophic lateral sclerosis | 1 (1.2%) |
| Anxiety disorder | 1 (1.2%) |
| Aortic valve stenosis | 1 (1.2%) |
| Apoplexy | 1 (1.2%) |
| Arterial hypertonia | 31 (38.3%) |
| Atrial fibrillation | 9 (11.1%) |
| Bronchial asthma | 4 (4.9%) |
| Carcinoma | 14 (17.3%) |
| Cardiac insufficiency | 3 (3.7%) |
| Chronic kidney disease | 1 (1.2%) |
| Chronic obstructive pulmonary disease (COPD) | 5 (6.2%) |
| Chronic pain syndrome | 1 (1.2%) |
| Community-acquired pneumonia (CAP) | 1 (1.2%) |
| Coronary vessel disease | 17 (21.0%) |
| Crohn's disease | 1 (1.2%) |
| Deep vein thrombosis | 1 (1.2%) |
| Dementia | 1 (1.2%) |
| Diabetes mellitus type 1 | 2 (2.5%) |
| Diabetes mellitus type 2 | 20 (24.7%) |
| Digitial dementia (Alzheimer type) | 1 (1.2%) |
| Dilated cardiomyopathy | 1 (1.2%) |
| Epilepsy | 2 (2.5%) |
| Glioblastoma | 1 (1.2%) |
| Hashimoto thyreoiditis | 2 (2.5%) |
| Hepatitis B | 2 (2.5%) |
| Hypercholesterolaemia | 1 (1.2%) |
| Hyperlipidemia | 1 (1.2%) |
| Hypertensive cardiac disease | 1 (1.2%) |
| Hyperuricaemia | 1 (1.2%) |
| Hypothyroidism | 1 (1.2%) |
| Hypothyroidism after thyroid glands surgery | 1 (1.2%) |
| Infantile brain damage | 1 (1.2%) |
| Infrarenal abdominal aortic aneurysm | 1 (1.2%) |
| Kidney transplantation | 2 (2.5%) |
| Lichen planus | 1 (1.2%) |
| Liver and kidney transplantation | 1 (1.2%) |
| Malignant facial tumour | 1 (1.2%) |
| Melanoma with cerebral metastasis (palliative) | 1 (1.2%) |
| Mesothelioma | 1 (1.2%) |
| Mitral valve insufficiency | 1 (1.2%) |
| Mixed dementia | 1 (1.2%) |
| Muscle dystrophy | 1 (1.2%) |
| Myotonic dystrophy type 2 | 2 (2.5%) |
| Neuroendocrine pankreatic tumour | 1 (1.2%) |
| Nicotine abuse | 2 (2.5%) |
| Non-ST-segment elevation myocardial infarction | 2 (2.5%) |
| None | 9 (11.1%) |
| Obesity | 4 (4.9%) |
| Obstructive sleep apnoea syndrome | 3 (3.7%) |
| Osteoporosis | 1 (1.2%) |
| Overweight | 1 (1.2%) |
| Parkinson's disease | 1 (1.2%) |
| Paroxysmal atrial fibrillation | 1 (1.2%) |
| Peripheral arterial disease | 5 (6.2%) |
| Posterior myocardial infarction | 1 (1.2%) |
| Pulmonary emphysema | 1 (1.2%) |
| Pulmonary fibrosis | 1 (1.2%) |
| Rheumatoid arthritis | 1 (1.2%) |
| Sarcoidosis type 2 with pulmonary involvement | 1 (1.2%) |
| Septic poisoning | 1 (1.2%) |
| Seronegative arthritis | 1 (1.2%) |
| Sjögren's syndrome | 1 (1.2%) |
| Spinal stenosis | 1 (1.2%) |
| Stroke with hemiparesis | 1 (1.2%) |
| Super infection (complicative) | 1 (1.2%) |
| Susac' s syndrome | 1 (1.2%) |
| Systemic Lupus erythematosus | 1 (1.2%) |
| Terminal kidney insufficiency | 1 (1.2%) |
| Transient ischaemic attack (complicative) | 1 (1.2%) |
| Tuberous sclerosis | 1 (1.2%) |
| Unknown | 1 (1.2%) |

^1^ n (%)
Note: Different underlying diseases distributed across 81 patients. Some patients had more than one underlying disease.

**Supplementary Table 4. Patient characteristics for swab samples.**

| **Characteristic** | **N = 45^1^** |
| --- | --- |
| **Sex** |  |
| female | 14 / 45 (31%) |
| male | 14 / 45 (31%) |
| NA | 17 / 45 (38%) |
| **Age** |  |
| Median (IQR) | 74.0 (52.0, 81.0) |
| Range | 35.0 - 91.0 |
| Unknown | 17 |
| **Grade** |  |
| asymptomatic | 2 / 45 (4.4%) |
| critical | 3 / 45 (6.7%) |
| mild | 6 / 45 (13%) |
| moderate | 8 / 45 (18%) |
| severe | 4 / 45 (8.9%) |
| NA | 22 / 45 (49%) |
| **Outcome** |  |
| Deceased | 5 / 45 (11%) |
| Survived | 23 / 45 (51%) |
| NA | 17 / 45 (38%) |
| **Remdesivir treated** |  |
| yes | 7 / 45 (16%) |
| no | 17 / 45 (38%) |
| NA | 21 / 45 (47%) |
| **Time between symptom onset and sample collection [days]** |  |
| Median (IQR) | 6.5 (4.0, 10.8) |
| Range | 0.0 - 25.0 |
| Unknown | 17 |

^1^ n / N (%)
NA: not available

**Supplementary Table 5. Oligonucleotides.**

| **Gene (target cell type)** | **Ensembl ID** | **Chromosome** | **Oligonucleotides for bisulfite sequencing** | | | **Demethyl-specific oligonucleotides for qPCR analysis** | | | | **As published in** |
| --- | --- | --- | --- | --- | --- | --- | --- | --- | --- | --- |
|  |  |  | Amplicon | Fw. (start - end) | Rev. (start - end) | Oligonucleotide | Genomic location | Sequence (5’-3’) | Conc. [µM] |  |
| GAPDH (Total cells) | ENSG00000111640 | Chr.12 | 1570 | 6534953 - 6534974 | 6535474 - 6535490 | Fw. | 6535212 - 6535233 | GGTTTTTGGTATTGTAGGTTTT | 1.5 | [2] |
|  |  |  |  |  |  | Rev. | 6535290 - 6535310 | CCAATTACAACATAACAACCA | 1.5 |  |
|  |  |  |  |  |  | Probe | 6535263 - 6535291 | TGTTTGGATGTTGTGTTTGTGGTAGAGTG | 0.25 |  |
| CD3G/D (Pan-T cells) | ENSG00000160654, ENSG00000167286 | Chr.11 | 1405 | 118343556 - 118343576 | 118343969 - 118343990 | Fw. | 118342917 - 118342938 | CCTAAACACTACCACATCTCAA | 1.5 |  |
|  |  |  | 1406 | 118342485 - 118342506 | 118342922 - 118342901 | Rev. | 118342971 - 118342994 | AGAAATTTAGTTGTTATGGTTTGT | 1.5 |  |
|  |  |  | 1408 | 118343987 - 118344009 | 118344437 - 118344458 | Probe | 118342949 - 118342975 | AAAAAACCATCAACCCCATAACACAAA | 0.25 |  |
| CD4 (T helper cells) | ENSG00000010610 | Chr.12 | 1255 | 6790192 - 6790213 | 6790582 - 6790603 | Fw. | 6790871 - 6790900 | CCCTACTCTTATAATAAACATTTTTATCAA | 4.5 | [3] |
|  |  |  | 2000 | 6790724 - 6790744 | 6791158 - 6791180 | Rev. | 6791046 - 6791072 | GAAATTATTTTTTGAGTGTTTTTAATG | 3.0 |  |
|  |  |  | 2001 | 6791141 - 6791166 | 6791539 - 6791560 | Probe | 6790997 - 6791022 | TGATTTTGAGGGTGGTGGTTATTTTG | 0.25 |  |
| CD8B (Cytotoxic T cells) | ENSG00000172116 | Chr.2 | 2007 | 86821232 - 86821253 | 86821695 - 86821673 | Fw. | 86821372 - 86821401 | GTGGTTAAGAAATTAATAGGAAAAAGAATG | 1.5 |  |
|  |  |  |  |  |  | Rev. | 86821463 - 86821483 | CTTCCCCACCACAATACAACA | 1.5 |  |
|  |  |  |  |  |  | Probe | 86821425 - 86821455 | TGTTTGTGAGGTATTTAGTTGATGGGAGTTT | 0.125 |  |
| LRP5 (B cells) | ENSG00000162337 | Chr.11 | 2249 | 68371460 - 68371481 | 68371926 - 68371947 | Fw. | 68371611 - 68371635 | AATATTACAACCATACACCCAACAA | 1.5 |  |
|  |  |  |  |  |  | Rev. | 68371720 - 68371748 | AAGTGATAGAATTTTATGTTTTTTTTATG | 1.5 |  |
|  |  |  |  |  |  | Probe | 68371662 - 68371688 | TTAGTTGAGGTGAGGTGTTTTGTTAGT | 0.25 |  |
| MVD (NK cells) | ENSG00000167508 | Chr.16 | 2674 | 88653882 - 88653903 | 88654300 - 88654320 | Fw. | 88654110 - 88654136 | GGTTTTGTGGTATTTTTATAGAGTAGT | 1.5 |  |
|  |  |  |  |  |  | Rev. | 88654172 - 88654190 | CCATATACACCCTCCTCAA | 1.5 |  |
|  |  |  |  |  |  | Probe | 88654135 - 88654159 | CCCTAAACCACCTCTTCCCCTACAC | 0.125 |  |
| LCN2 (Neutrophils) | ENSG00000148346 | Chr.9 | 1730 | 1793657 - 1793677 | 1793816 - 1793838 | Fw. | 128149258 - 128149278 | ACCAAAAATACAACACTTCAA | 1.5 |  |
|  |  |  |  |  |  | Rev. | 128149353 - 128149375 | GGTAATTGTTAGTAATTTTTGTG | 1.5 |  |
|  |  |  |  |  |  | Probe | 128149289 - 128149309 | CACTCTCCCCATCCCTCTATC | 0.15 |  |
| FOXP3 (regulatory T cells) | ENSG00000049768 | Chr.X | 772 | 49260578 - 49260598 | 49261028 - 49261049 | Fw. | 49260762 - 49260789 | GTTTTTGATTTGTTTAGATTTTTTTGTT | 1.5 | [2,4] |
|  |  |  |  |  |  | Rev. | 49260826 - 49260850 | CCTCTTCTCTTCCTCCATAATATCA | 1.5 |  |
|  |  |  |  |  |  | Probe | 49260799 - 49260821 | ATGGTGGTTGGATGTGTTGGGTT | 0.25 |  |
| CBX6 (memory B cells) | ENSG00000183741 | Chr.22 | 3005 | 38863396 - 38863420 | 38863871 - 38863892 | Fw. | 38863431 - 38863450 | GGAAAGTAGTAAGGGTGGAT | 1.5 | unpublished |
|  |  |  |  |  |  | Rev. | 38863510 - 38863527 | CCCTCTCTAATACCCCCA | 1.5 |  |
|  |  |  |  |  |  | Probe | 38863485 - 38863512 | TTGATTTGTGTAAGTGTGTGGGAGGTGG | 0.125 |  |
| C7orf50 (naïve B cells) | ENSG00000146540 | Chr.7 | 4288 | 1025980 - 1026001 | 1026457 - 1026478 | Fw. | 1026198 - 1026226 | CTTAAACATATAAAC[I]CTATCTAACAA | 1.5 |  |
|  |  |  |  |  |  | Rev. | 1026248 - 1026277 | GTTTTATTTTTAATTGGTTTAATAGTAGTG | 1.5 |  |
|  |  |  |  |  |  | Probe | 1026227 - 1026250 | AAG+AAATGAGG+AG+TGT+TGTG | 0.125 |  |

Note: List shows genomic localisation of bisulfite-sequencing pimers (final concentration is 0.5 µM) as well as bisulfite- and demethylation-specific primer and hydrolysis probe sequences used for qPCR. Ensembl release 102 (Genome build: GRch 38.102) was used for determination of oligonucleotide positions. [I] indicates the universal nucleotide inosine (2’Deoxyinosine). Sequence modification by a locked nucleic acid (LNA) is indicated by a “+” before the affected nucleotide (e.g. +T).

**Supplementary Table 6. Result table to figure 2.**

| **Marker** | **Healthy cohort** | | | **Bochum cohort** | | | **Valencia cohort** | | |
| --- | --- | --- | --- | --- | --- | --- | --- | --- | --- |
|  | **total, N = 113^1^** | **female, N = 59^1^** | **male N = 54^1^** | **total, N = 75^1^** | **female, N = 40^1^** | **male N = 35^1^** | **total, N = 22^1^** | **female, N = 6^1^** | **male N = 16^1^** |
| Neutrophils [%] | 56.0 (49.7, 64.0) | 60.2 (52.7, 65.9) | 54.1 (48.5, 58.2) | 64.9 (56.0, 73.8) | 61.9 (55.7, 74.4) | 65.9 (56.2, 73.0) | 78.4 (67.9, 83.5) | 76.6 (65.7, 78.7) | 80.7 (71.6, 86.7) |
| T cells [%] | 23.5 (19.5, 29.0) | 23.4 (19.5, 27.6) | 24.1 (19.4, 29.1) | 11.4 (8.1, 17.8) | 13.5 (7.8, 19.5) | 11.1 (8.4, 16.4) | 5.1 (4.4, 8.1) | 6.8 (5.1, 10.0) | 4.9 (4.2, 6.7) |
| T helper cells [%] | 16.4 (13.2, 19.9) | 16.7 (12.5, 19.1) | 16.0 (13.3, 20.6) | 7.7 (4.7, 12.0) | 7.8 (4.3, 12.8) | 7.2 (5.2, 10.4) | 3.4 (2.4, 4.3) | 3.9 (3.3, 7.4) | 2.8 (2.4, 4.2) |
| Cytotoxic T cells [%] | 7.2 (5.5, 9.4) | 6.9 (5.5, 9.0) | 7.2 (5.8, 9.5) | 4.1 (2.4, 6.7) | 4.9 (2.5, 6.8) | 3.5 (2.4, 5.4) | 2.2 (1.5, 3.5) | 2.2 (1.8, 3.5) | 2.1 (1.3, 3.5) |
| B cells [%] | 4.9 (3.8, 6.3) | 4.5 (3.5, 6.0) | 5.1 (3.9, 6.6) | 2.3 (1.1, 3.8) | 2.3 (1.3, 3.5) | 2.1 (1.0, 3.8) | 2.0 (1.6, 3.8) | 3.2 (1.9, 4.4) | 1.9 (1.5, 3.7) |
| NK cells [%] | 5.9 (5.1, 6.8) | 5.7 (4.9, 6.6) | 6.2 (5.3, 7.1) | 6.0 (4.6, 7.1) | 5.9 (4.5, 6.9) | 6.0 (4.7, 7.1) | 5.3 (4.7, 6.9) | 6.1 (4.7, 7.8) | 5.3 (5.0, 6.2) |
| Tregs [%] | 1.3 (1.0, 1.6) | 1.4 (1.1, 1.6) | 1.2 (1.0, 1.5) | 0.8 (0.5, 1.0) | 0.9 (0.8, 1.5) | 0.5 (0.3, 0.6) | 0.5 (0.3, 0.8) | 1.0 (0.9, 1.0) | 0.4 (0.3, 0.6) |
| memory B cells [%] | 1.5 (1.3, 1.9) | 1.6 (1.3, 2.0) | 1.5 (1.3, 1.8) | 1.3 (0.9, 1.8) | 1.2 (0.9, 1.8) | 1.3 (0.9, 1.9) | 1.3 (1.0, 1.5) | 1.4 (1.2, 1.8) | 1.3 (1.0, 1.5) |
| naive B cells [%] | 2.1 (1.5, 2.9) | 1.9 (1.4, 2.5) | 2.4 (1.6, 3.0) | 1.3 (0.7, 1.8) | 1.3 (0.8, 1.8) | 1.3 (0.7, 1.8) | 1.2 (0.7, 2.6) | 1.8 (0.7, 3.0) | 1.2 (0.8, 1.8) |
| LNR | 0.63 (0.47, 0.76) | 0.58 (0.46, 0.71) | 0.68 (0.56, 0.85) | 0.3 (0.2, 0.5) | 0.34 (0.20, 0.58) | 0.34 (0.21, 0.43) | 0.19 (0.15, 0.24) | 0.24 (0.18, 0.33) | 0.16 (0.14, 0.19) |
| **Marker** | **p^2^ value** | | | | | | | | |
|  | **Healthy cohort, male (N = 54)/ female (N = 59)** | | | **Bochum cohort, male (N = 35)/ female (N = 40)** | | | **Valencia cohort, male (N = 16)/ female (N = 6)** | | |
| Neutrophils [%] | 0.006 | | | 0.6 | | | 0.2 | | |
| T cells [%] | 0.6 | | | 0.5 | | | 0.09 | | |
| T helper cells [%] | 0.7 | | | 0.6 | | | 0.1 | | |
| Cytotoxic T cells [%] | 0.5 | | | 0.3 | | | 0.4 | | |
| B cells [%] | 0.2 | | | 0.9 | | | 0.4 | | |
| NK cells [%] | 0.04 | | | 0.7 | | | 0.6 | | |
| Tregs [%] | 0.05 | | | < 0.0001 | | | 0.002 | | |
| memory B cells [%] | 0.9 | | | 0.5 | | | 0.4 | | |
| naive B cells [%] | 0.04 | | | 0.7 | | | 0.9 | | |
| LNR | 0.01 | | | 0.6 | | | 0.05 | | |
| **Marker** | **p^2^ value** | | | | | | | | |
|  | **Healthy (N = 113)/ Bochum (N = 75)** | | | **Healthy (N = 113)/ Valencia (N = 22)** | | | **Bochum (N = 75)/ Valencia (N = 22)** | | |
|  | **total** | **female** | **male** | **total** | **female** | **male** | **total** | **female** | **male** |
| Neutrophils [%] | 0.0001 | 0.1 | < 0.0001 | < 0.0001 | 0.02 | < 0.0001 | 0.0002 | 0.1 | 0.002 |
| T cells [%] | < 0.0001 | < 0.0001 | < 0.0001 | < 0.0001 | < 0.0001 | < 0.0001 | < 0.0001 | 0.04 | < 0.0001 |
| T helper cells [%] | < 0.0001 | < 0.0001 | < 0.0001 | < 0.0001 | 0.0001 | < 0.0001 | < 0.0001 | 0.05 | < 0.0001 |
| Cytotoxic T cells [%] | < 0.0001 | 0.0002 | < 0.0001 | < 0.0001 | 0.0001 | < 0.0001 | 0.0004 | 0.04 | 0.02 |
| B cells [%] | < 0.0001 | < 0.0001 | < 0.0001 | < 0.0001 | 0.2 | < 0.0001 | 0.7 | 0.3 | 0.9 |
| NK cells [%] | 0.5 | 0.8 | 0.3 | 0.2 | 0.7 | 0.04 | 0.7 | 0.6 | 0.3 |
| Tregs [%] | < 0.0001 | 0.001 | < 0.0001 | < 0.0001 | 0.009 | < 0.0001 | 0.05 | 1 | 0.3 |
| memory B cells [%] | 0.01 | 0.02 | 0.2 | 0.07 | 0.7 | 0.05 | 0.9 | 0.3 | 0.6 |
| naive B cells [%] | < 0.0001 | 0.0002 | < 0.0001 | 0.01 | 0.8 | 0.004 | 0.5 | 0.6 | 0.6 |
| LNR | < 0.0001 | < 0.0001 | < 0.0001 | < 0.0001 | 0.0003 | < 0.0001 | 0.0004 | 0.2 | < 0.0001 |

^1^Median (IQR)

^2^Wilcoxon rank sum test

Abbreviations: lymphocyte-to-neutrophile ratio (LNR)

**Supplementary Table 7. Differences between disease stages between both disease cohorts.**

| **Assay** | **Bochum-to-Valencia** | | |
| --- | --- | --- | --- |
|  | **p value^1^** | | |
|  | **moderate-to-moderate** | **severe-to-severe** | **critical-to-critical** |
| T cells | 0.7 | 0.004 | 0.05 |
| T helper cells | 0.4 | 0.004 | 0.03 |
| Cytotoxic T cells | 0.2 | 0.03 | 0.003 |
| B cells | 0.3 | 0.01 | 1.0 |
| NK cells | 1.0 | 0.2 | 0.4 |
| Tregs | 0.4 | 0.1 | 1.0 |
| Neutrophils | 0.4 | 0.002 | 0.02 |
| Memory B cells | 0.1 | 0.03 | 0.7 |
| Naive B cells | 0.08 | 0.07 | 0.8 |
| LNR | 0.8 | 0.02 | 0.1 |

^1^Kolmogorow-Smirnow
Note: p values are calculated by using all time points.
Abbreviations: Lymphocyte-to-Neutrophile ratio (LNR)

**Supplementary Table 8. Result table to figure 3.**

| **Marker** | **Prognosis** | **Median (IQR)** | **p value^1^** |  |
| --- | --- | --- | --- | --- |
|  |  |  |  |  |
| B cells [%] | Good | 2.2 (1.2, 3.3) | 0.1 |  |
|  | Poor | 0.8 (0.6, 2.3) |  |  |
| T cells [%] | Good | 15.7 (10.8, 19.7) | 0.017 |  |
|  | Poor | 8.1 (5.4, 10) |  |  |
| T helper cells [%] | Good | 9.3 (6.8, 12.8) | 0.019 |  |
|  | Poor | 5.3 (4, 6.9) |  |  |
| Cytotoxic T cells [%] | Good | 5.3 (3.3, 7) | 0.079 |  |
|  | Poor | 2.9 (1.5, 3.7) |  |  |
| Tregs [%] | Good | 0.8 (0.6, 1.4) | 0.18 |  |
|  | Poor | 0.6 (0.4, 0.8) |  |  |
| memory B cells [%] | Good | 1.3 (0.9, 1.7) | 0.86 |  |
|  | Poor | 0.9 (0.8, 1.8) |  |  |
| naive B cells [%] | Good | 1.3 (0.9, 1.8) | 0.0024 |  |
|  | Poor | 0.4 (0.4, 0.9) |  |  |
| Neutrophils [%] | Good | 56.3 (52.2, 66.9) | 0.0027 |  |
|  | Poor | 71.2 (62.4, 77.9) |  |  |
| NK cells [%] | Good | 6.5 (5.1, 9.1) | 0.43 |  |
|  | Poor | 5.9 (4.2, 6.8) |  |  |
| LNR | Good | 0.4 (0.3, 0.6) | 0.0054 |  |
|  | Poor | 0.2 (0.2, 0.3) |  |  |

^1^Wilcoxon rank sum test

Abbreviations: lymphocyte-to-neutrophile ratio (LNR)

**Supplementary Table 9. Prognostic performance for investigated epigenetic markers.**

| **Marker** | **AUC (95% CI)** | **Specificity** | **Sensitivity** | **Accuracy** | **Optimal Threshold** |
| --- | --- | --- | --- | --- | --- |
| T cells | 0.77 (0.59-0.96) | 0.81 | 0.78 | 0.80 | 10.2% cells |
| Naive B cells | 0.84 (0.71-0.98) | 0.67 | 1.00 | 0.75 | 1.05% cells |
| Neutrophils | 0.83 (0.68-0.97) | 0.67 | 0.89 | 0.72 | 58.6% cells |
| LNR | 0.81 (0.63-0.98) | 0.88 | 0.67 | 0.83 | 0.21 |
| Combined | 0.93 (0.85-1.00) | 0.67 | 0.88 | 0.83 | NA |

Note: Good prognosis was encoded as 0 and poor prognosis as 1. For all markers the obtained threshold is used to classify values above as 1. Resulting AUC (area under curve) including the 95% confidence interval (CI) are shown. Sensitivity, Specificity, Accuracy and Optimal threshold were determined by Youden's J statistic. “Combined” corresponds to the multivariate analysis of the four markers for T cells, naive B cells, neutrophils, and the lymphocyte-to-neutrophile ratio (LNR). Multivariate analysis was performed using a logistic regression, predicting the outcome based on a choice of marker after univariate analysis. For the prediction in the multivariate model a threshold of 0.5 was used.

**Supplementary Table 10. Result table to figure 5.**

| **Marker** | **Substrate** | | | | | **p value**^2^ | | |
| --- | --- | --- | --- | --- | --- | --- | --- | --- |
|  | **Blood_HD, N = 113^1^** | **Swab_HD, N = 69^1^** | **Swab_COVID** | | | **Blood_HD/ Swab_HD** | **Swab_HD / Swab_COVID** | **mild & moderate/ severe & critical** |
|  |  |  | **All Samples, N = 45^1^** | **mild & moderate, N = 27^1^** | **severe & critical, N = 17^1^** |  |  |  |
| B cells [%] | 4.9 (3.8, 6.3) | 1.3 (0.5, 2.4) | 0.5 (0.3, 1.1) | 0.8 (0.4, 1.7) | 0.3 (0.2, 0.4) | < 0.0001 | 0.002 | 0.09 |
| T cells [%] | 20.4 (16.7, 25.8) | 3.6 (2.3, 6.9) | 2.2 (0.9, 3.7) | 1.8 (0.9, 3.6) | 0.9 (0.5, 1.9) | < 0.0001 | 0.0009 | 0.08 |
| memory B cells [%] | 1.5 (1.3, 1.9) | 0.6 (0.6, 0.9) | 0.8 (0.5, 1.3) | 1.2 (1.0, 1.8) | 1.4 (0.6, 1.7) | < 0.0001 | 0.6 | 0.8 |
| naive B cells [%] | 2.1 (1.5, 2.9) | 1.3 (0.8, 1.9) | 1.3 (0.8, 2.2) | 1.8 (1.1, 2.5) | 0.6 (0.3, 0.8) | 0.002 | 0.8 | 0.03 |
| Nk cells [%] | 3.0 (2.4, 4) | 0.9 (0.7, 1.5) | 0.7 (0.4, 1.7) | 1.2 (0.6, 1.5) | 0.4 (0.3, 1.0) | < 0.0001 | 0.3 | 0.05 |

^1^Median (IQR)

^2^Wilcoxon rank sum test

**Supplementary Table 11. Immune cell counts by disease stage.**

| **Variable** | **Bochum, N = 165 samples** | | | **Valencia, N = 90 samples** | | |
| --- | --- | --- | --- | --- | --- | --- |
|  | **moderate, N = 108^1^** | **severe, N = 40^1^** | **critical, N = 17^1^** | **moderate, N = 16^1^** | **severe, N = 41^1^** | **critical, N = 33^1^** |
| Neutrophile [%] | 60.7 (53.2, 69.5) | 64.3 (54.4, 72.9) | 71.0 (65.1, 73.9) | 63.2 (59.2, 74.6) | 77.6 (66.0, 85.5) | 82.0 (69.8, 86.1) |
| T cells [%] | 14.3 (9.3, 19.5) | 10.2 (5.5, 14.1) | 8.3 (6.0, 15.3) | 12.4 (6.0, 18.3) | 5.4 (4.0, 8.2) | 5.6 (3.7, 7.3) |
| T helper cells [%] | 9.0 (5.7, 12.9) | 6.3 (3.8, 7.9) | 4.8 (3.9, 8.1) | 7.5 (3.3, 11.5) | 4.0 (2.5, 4.9) | 3.3 (2.1, 4.3) |
| Cytotoxic T cells [%] | 4.8 (3.1, 6.9) | 3.1 (2.0, 6.2) | 3.1 (2.0, 4.7) | 3.0 (2.0, 7.8) | 2.0 (1.3, 3.4) | 1.7 (1.3, 3.3) |
| B cells [%] | 2.3 (1.1, 3.8) | 1.5 (0.7, 2.8) | 2.1 (1.1, 4.8) | 3.3 (2.3, 7.8) | 2.7 (1.6, 4.3) | 2.6 (1.0, 3.5) |
| NK cells [%] | 6.0 (4.7, 7.2) | 5.8 (4.5, 7.1) | 4.2 (4.0, 5.2) | 5.6 (4.4, 6.9) | 5.2 (4.7, 6.8) | 4.9 (4.2, 5.3) |
| Tregs [%] | 0.9 (0.6, 1.3) | 0.8 (0.5, 1.0) | 0.4 (0.3, 0.6) | 0.8 (0.6, 1.1) | 0.5 (0.3, 0.8) | 0.4 (0.2, 0.7) |
| Memory B cells [%] | 1.4 (0.9, 1.9) | 1.1 (0.7, 1.5) | 1.3 (0.8, 1.5) | 1.8 (1.1, 3.6) | 1.3 (1.0, 2.0) | 1.1 (0.8, 1.6) |
| Naive B cells [%] | 1.2 (0.7, 1.9) | 0.9 (0.5, 1.4) | 1.3 (0.7, 1.6) | 2.1 (1.0, 3.8) | 1.6 (0.9, 2.6) | 1.3 (0.7, 2.4) |
| LNR | 0.37 (0.25, 0.56) | 0.29 (0.14, 0.43) | 0.26 (0.15, 0.33) | 0.38 (0.19, 0.60) | 0.19 (0.15, 0.28) | 0.16 (0.12, 0.22) |
| **Variable** | **p^2^ value** | | | **p^2^ value** | | |
|  | **moderate to severe** | **severe to critical** | **moderate to critical** | **moderate to severe** | **severe to critical** | **moderate to critical** |
| Neutrophile [%] | 0.2 | 0.09 | 0.003 | 0.02 | 0.5 | 0.003 |
| T cells [%] | 0.007 | 0.7 | 0.008 | 0.003 | 0.3 | 0.004 |
| T helper cells [%] | 0.005 | 0.5 | 0.007 | 0.008 | 0.2 | 0.007 |
| Cytotoxic T cells [%] | 0.03 | 1 | 0.04 | 0.02 | 0.9 | 0.01 |
| B cells [%] | 0.02 | 0.3 | 0.9 | 0.2 | 0.4 | 0.07 |
| NK cells [%] | 0.6 | 0.003 | 0.0006 | 1 | 0.01 | 0.09 |
| Tregs [%] | 0.09 | 0.004 | < 0.0001 | 0.08 | 0.07 | 0.01 |
| Memory B cells [%] | 0.02 | 0.3 | 0.4 | 0.1 | 0.1 | 0.01 |
| Naive B cells [%] | 0.05 | 0.5 | 0.6 | 0.3 | 0.4 | 0.2 |
| LNR | 0.008 | 0.4 | 0.002 | 0.008 | 0.2 | 0.003 |

^1^Median (IQR)
^2^Wilcoxon rank sum test
Abbreviations: Lymphocyte-to-neutrophile ratio (LNR)

**References**

1. Lewin J, Schmitt AO, Adorján P, Hildmann T, Piepenbrock C. Quantitative DNA methylation analysis based on four-dye trace data from direct sequencing of PCR amplificates. Bioinformatics. **2004**; 20(17):3005–3012.

2. Sehouli J, Loddenkemper C, Cornu T, et al. Epigenetic quantification of tumor-infiltrating T-lymphocytes. Epigenetics. **2011**; 6(2):236–246.

3. Baron U, Werner J, Schildknecht K, et al. Epigenetic immune cell counting in human blood samples for immunodiagnostics. Sci Transl Med [Internet]. **2018**; 10(452). Available from: http://stm.sciencemag.org/content/10/452/eaan3508.abstract

4. Baron U, Floess S, Wieczorek G, et al. DNA demethylation in the human FOXP3 locus discriminates regulatory T cells from activated FOXP3+ conventional T cells. Eur J Immunol. **2007**; 37(9):2378–2389.
